# Combined Approach to Evaluate Hydrate Slurry Transport Properties through Wetting and Flow Experiments

**DOI:** 10.1021/acsomega.2c05773

**Published:** 2023-01-09

**Authors:** Martin Fossen, Stephan Hatscher, Luis Ugueto

**Affiliations:** † Multiphase Flow, Department of Process Technology, SINTEF AS, 0314Oslo, Norway; ‡ Wintershall DEA Norge AS, Jåttåflaten 27, 4020Stavanger, Norway

## Abstract

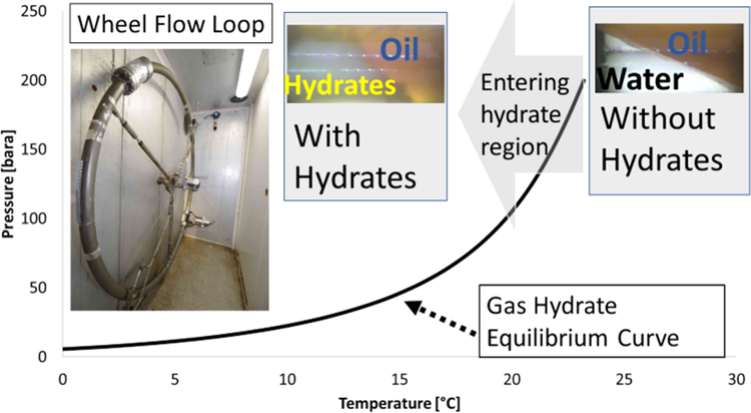

A condensate oil system was evaluated with respect to its hydrate properties by two experimental methods, namely, the wetting index (WI) procedure and a flow loop called the wheel flow loop. The WI was used to initially indicate the efficiency of a gas hydrate antiagglomerant (AA), while the wheel flow loop was used for evaluating the transport properties of systems without and with AA. The results provide new insight into the effect of water cut and flow properties on the risk of hydrate plugging. The test case used in the study was a relevant field from the Vega gas condensate asset on the Norwegian continental shelf. This asset is currently producing using continuous monoethylene glycol (MEG) injection as a hydrate prevention philosophy. The wettability of the hydrate particles was determined for uninhibited, underinhibited (10% MEG), and AA-inhibited systems, and the results indicated favorable wettability of the AA-protected system by changing the emulsion inversion point to higher water cuts. Furthermore, the wettability data were then confirmed by flow tests utilizing SINTEF’s wheel flow loop. Moreover, both uninhibited and underinhibited systems led to plugging upon hydrate formation, indicating the need for optimized AA concentrations for a given fluid system and water cut. The overall results show that the WI combined with the wheel flow loop or similar equipment is an effective method for better selection and description of the plugging potential and transport properties for gas hydrate systems.

## 1 Introduction

Prediction of the susceptibility of a natural gas-based system to form clathrate gas hydrates^1^ in the presence of water is a major task when developing assets or understanding their flow assurance challenges during production of natural gas, condensates, or crude oils.^2−7^ A range of different test methods for experimental verification of hydrate formation pressure, temperature, and kinetics have been developed and are regularly used to study the properties of gas hydrates like the ones outlined in the recent reviews by Salmin, Estanga, and Koh, focusing on antiagglomerant screening techniques,^8^ and Almashwali et al. on gas hydrates in oil-dominated systems.^6^ The methods mentioned in the literature focus on direct determination of the plugging potential for both uninhibited and inhibited gas hydrate systems and determination of the inhibition degree as well as delay in hydrate formation as a function of subcooling. Moreover, a method developed to determine the wettability of gas hydrate particles and indirectly indicate the potential to form or not form hydrate plugs is described by Høiland et.al.^9^ This method is called the “wetting index” (WI). Further details of the development and use of this WI are given elsewhere in the literature.^3,10,11^ The WI method exploits the concept postulated by Bancroft for surfactants in 1913, which states that “a hydrophile colloid will tend to make water the dispersion phase while a hydrophobic colloid will tend to make the water the disperse phase”.^12^ Thus, a hydrate particle with a hydrophobic surface will not easily be attracted or agglomerated with another hydrate particle, since water-bridging will be weak compared to water-wet particles.

Gas hydrates constitute the largest problem by an order of magnitude relative scaling, wax and asphaltene precipitation.^1^ Therefore, the understanding of their properties and ability to predict possible risks of plugging is one of the main flow assurance challenges of oil and gas producer operating fields where conditions favor hydrate formation.^3,4,7,13−17^ If not properly managed, gas hydrates may form wall deposits, lumps, plugs, or thick slurries that can block pipelines and process equipment thus with potentially severe consequences.^3^ The traditional, conservative, and safest approach to mitigate gas hydrate challenges consists of rather costly methods such as direct electrical heating (DEH), often combined with insulation, or the use of large volumes of thermodynamic hydrate inhibitors (THIs),^16,18^ usually either methanol or glycol. Another option is to design the system with sufficient freedom to be able to reduce the pressure to bring the system outside the hydrate region when required (typically upon shutdown). In the case of the formation of a hydrate plug, the most common solution is to depressurize the pipeline, preferably on both sides of the hydrate plug simultaneously. Nevertheless, due to the complexities and costs of traditional hydrate management, cheaper and safer methods for gas hydrate avoidance or control are continuously looked for.

An alternative hydrate mitigation strategy to the ones mentioned above is the use of low-dosage gas hydrate inhibitors (LDHIs).^3,7,13−15,18,19^ LDHIs are divided into kinetic hydrate inhibitors (KHIs) or antiagglomerant (AA) hydrate inhibitors, and both types push the limits for safe operation at lower potential costs. One of the main cost savings when using LDHIs is the avoidance of CAPEX-intensive methanol or glycol regeneration facilities.^19^ In addition, THIs often need to be used at 20–50 vol % relative to the water phase,^20^ which is an order of magnitude higher than the volumes required with LDHIs.

In light of the challenges of the Vega asset, described below, the objective of the current work was to experimentally evaluate alternative flow assurance approaches for the gas condensate system utilizing the wetting index method and a wheel flow loop as described elsewhere in the literature.^13,21,22^ AAs are designed to render the gas hydrate particles oil-wet when formed, thus avoiding agglomeration and plugging.^9,11,23^ We present in this work experimental data on the wettability and transportability of the Vega fluid under different conditions, namely, uninhibited, underinhibited (10% MEG), and with an AA present. The results from the experiments are further discussed in the context of a potential field application in Vega assets and in gas condensate fields in general. A layout of the field and further details of the first decade of operation is described elsewhere by Hatscher et al.^24^ Mitigation strategies to avoid either formation of hydrate plugs or plugging due to hydrates formed have been evaluated. The strategies involve the use of reactive depressurization, injection of limited amounts of MEG, or the use of antiagglomerants.

Reactive depressurization is used to prevent the system from entering the hydrate region, during both shutdown and restart. However, both events would expose the asset to potential hydrate formation and even blockage. The second method, MEG injection, would allow for additional subcooling to be tolerated. In essence, the same risks appear as described above for the “uninhibited case,” but the operational window would be enlarged. Still, it has been reported^13^ that underinhibited gas condensate systems might face gelation of hydrates and the appearance of a more viscous phase, easily blocking flowlines. Finally, the use of an LDHI of the antiagglomerant type would allow the production of the field “as is” upon shutdowns and allow the formation of gas hydrates while protecting the system from plugging. An AA-protected system will disperse the gas hydrate particles into the liquid hydrocarbon phase, preventing deposition of hydrates to the pipe wall or formation of hydrate lumps, which can grow and eventually block the pipeline. However, even with a working AA, there is still a danger of blocking the pipeline if the slurry viscosity becomes too high to be transportable, a risk that increases with increasing water cut. Both the pressure drop of an increased viscosity slurry and the handling capacity of the receiving units of the host facilities would need to be evaluated if the AA of relevance can be used. Thus, understanding the flow behavior of the slurry formed is therefore very important for defining the operational mode.^25^

## 2 Materials and Methods

### 2.1 Water Chemistry

The water phase given in Table 1 represented the composition of the relevant field and was also used when producing the hydrate curve given in Figure 8. In addition to salt ions, 300 ppm (weight) of an imidazoline-type corrosion inhibitor relative to the water phase was used, and the pH was adjusted to 4.8 by addition of HCl to match the pH of the Vega water phase. Moreover, the AA was chosen over kinetic hydrate inhibitors for this study, since it meets requirements regarding high subcooling, less sensitivity to shut-in durations, better thermal stability at elevated temperatures, good compatibility with the corrosion inhibitor, and topside treatment conditions. Regarding environmental issues, the selected AA was deemed an improvement to other AA alternatives.

**Table 1:** Brine Composition as Used in the WI Tests

component	amount [wt %]
NaCl	0.68
CaCl_2_	0.15
NaHCO_3_	0.01
sodium acetate	0.02
acetic acid	5.0 × 10^–4^
tap water	99.14
total	100
corrosion inhibitor	300 ppm

### 2.2 Fluid Data

The oil phase used was a condensate from the Vega asset and was provided in closed metal jerry cans and employed as received. The saturate, aromatic, resin, and asphaltene (SARA) composition and wax content of the condensate are given in Table 2. The pour point was measured to be below 0 °C.

**Table 2:** Oil Composition

fraction	amount [wt %]
saturates	79.3
aromatics	19.8
resins	0.9
asphaltenes	<0.1
wax content	<0.5

Furthermore, the gas composition used to calculate the hydrate curve was based on the flash of the gas phase from the reservoir fluid and simplified to the composition in Table 3. The actual composition for these experiments deviated slightly after adjusting to match the hydrate curve of the reservoir composition.

**Table 3:** Gas Compositions in Mol % for the Reservoir Conditions and the Actual Mole Composition of the Gas Filled to the Wheel Flow Loop

component	reservoir fluid [mol %]	actual [mol %]
N_2_	0.8	0.0
CO_2_	2.9	3.4
CH_4_ (methane)	83.2	77.7
C_2_H_6_ (ethane)	7.7	10.7
C_3_H_8_ (propane)	4.7	7.6
iC_4_(iso-butane)	0.7	0.7
total	100.0	100.0

To determine the amount of the condensate, gas components, and the brine phase to be filled to the experimental setup, PVTSim Nova 4.1 by Calsep was used for thermodynamic calculations. The compositions in Tables 3 and 4 were designed so that the liquid volume fraction in the flow loop was 40% at a given water cut and that the hydrate curve of the composition filled to the wheel was comparable to the field conditions, ensuring comparable driving forces and subcooling for hydrate formation. The above criteria were met by adjusting the gas composition, resulting in a slight deviation from the field composition as shown in Table 3. In Table 4, the target and actual mass of the condensate, brine, and gas components for tests 3–6 are given.

**Table 4:** Detailed Filling Composition for AA Wheel Tests 3–6

compound	target [g]	actual [g]
water	3149	3214
oil	1420	1396
iC4	17	16
C3	136	139
CO_2_	54	63
C2	122	134
C1	504	520
AA	47	47
corrosion inhibitor	300 ppm(m) weight on water	300 ppm(m) weight on water

### 2.3 Wetting Index Autoclave Experiments

**Figure 1:**
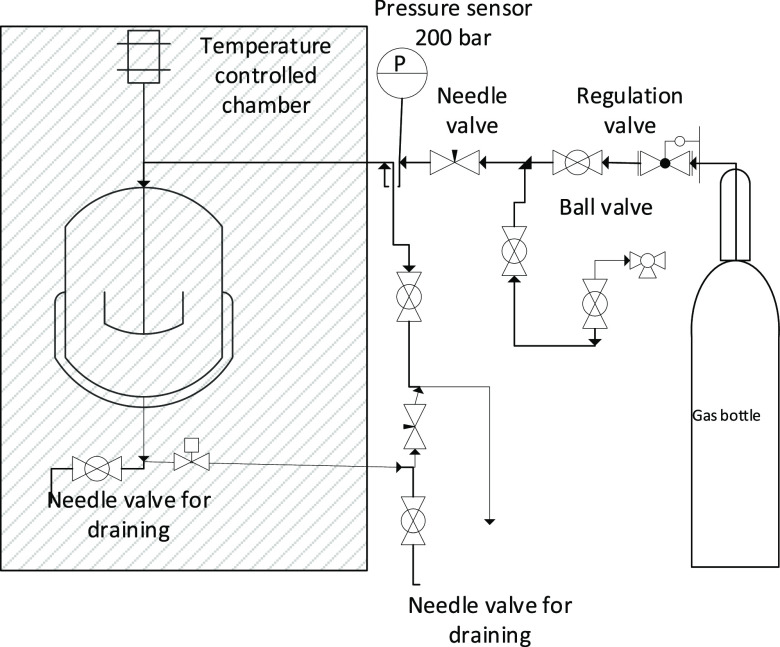
Schematic drawing of the autoclave setup. The stirred cell sits in a temperature-controlled chamber (left) and was filled with oil and water before being pressurized with a premixed gas phase from the gas bottle (right).

**Figure 2:**
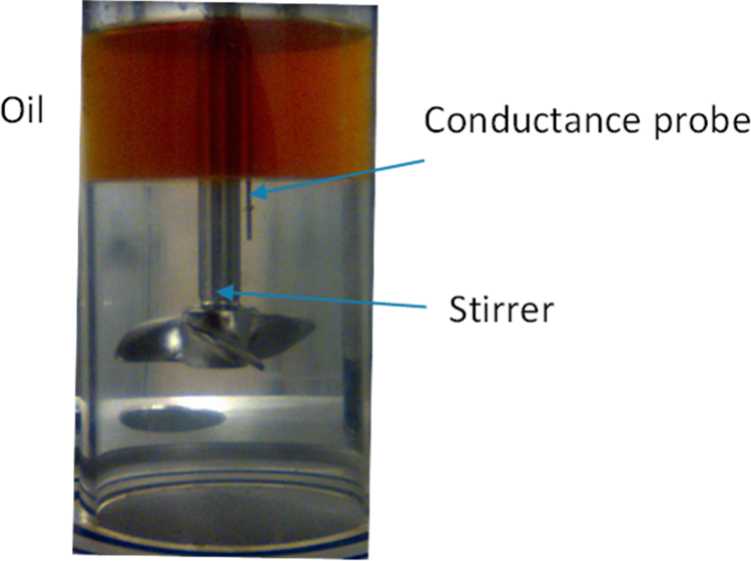
Picture of the autoclave used for wetting index tests, indicating the oil phase above the water phase (transparent), when not stirred, and the stirrer and the conductance probe position.

The WI method determines the change in the inversion point from oil continuous to the point where the oil no longer manages to keep the water droplets dispersed. The change in the emulsion inversion point is used to calculate the degree of oil or water wetting of gas hydrate particles.^26,27^ Moreover, the wetting index experiments were performed in a high-pressure autoclave at SINTEF’s Multiphase Flow Laboratory at Tiller in Norway. It consisted of a 380 mL poly(methyl methacrylate) (PMMA) tube with an outer diameter of 120 mm and an inner diameter (ID) of 50 mm. The PMMA tube was placed between two flanges (316L PN400). The connected stirrer (Parr magnetic stirrer) was used to mix the phases, ensuring fully dispersed liquid–liquid systems. The temperature was measured by positioning a PT-100 element in the liquid phase, and the pressure was measured using a Fujii ATEX 0074 transmitter. A probe (metal rod SS316) inserted from the top was used for measuring the conductance in the liquid phase. The entire autoclave setup was placed inside a temperature-controlled chamber. The measurement of the conductance in the liquid phase was the main information needed to determine the wetting index. High conductance indicated a water continuous system and low conductivity indicated an oil continuous system. The gas phase used was a 92/8 mol % methane/propane mixture (provided by Linde Gas AS). Pressurization of the cell with this gas was done manually by opening a valve from the gas bottle to the autoclave. For visual observation, a video camera was used for monitoring and capturing videos from the cell. Schematics of the setup are given in Figure 1, while a picture of the cell filled with water and condensate is shown in Figure 2. The applied voltage and measured current were used to determine the conductance of the fluid system (liquid phase). Conductance is the inverse of resistivity and is more convenient to use than resistivity for electrolytic solutions. The resistivity *R* is related to the alternating current *I* and the applied voltage *E*, as shown in eq 1, and the conductance *L* is thus defined as the unit Ω^–1^ or Siemens (S),^28^ as given in eq 2.
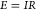
1
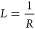
2

### 2.4 Wetting Index Principle and Test Procedure

The principle for measuring the wetting index was first developed by Høiland et al.^9^ and is also described elsewhere.^11,17^ The wetting index is based on the fact that gas hydrate particles have a water-wet surface. By either adding synthetic AAs, or through the existence of natural antiagglomerants, it is possible to modify the surface wettability from water-wet to oil-wet. To determine if the hydrate particles are oil-wet, a range of tests at different water cuts are necessary. The testing is done by selecting a starting water cut (i.e 50%) and then pressurizing with the hydrocarbon gas phase. When the pressure is increased and the system is cooled to well within the hydrate region while stirring, hydrate formation eventually occurs. From the conductance, the continuous phase can be determined (i.e., being either oil or water continuous). Since the procedure includes cooling into the hydrate region while measuring the conductance, one measures the nature of the continuous phase both with and without gas hydrates present in the same run for a single WC. Ultimately, after testing sufficient water cuts, one will determine the inversion points needed to calculate the WI, providing a single number between −1 and +1 for the given fluid system. A negative WI value indicates water-wet hydrate particles, while a positive number indicates oil-wet hydrate particles. A value of 0 indicates that the hydrate particles did not change the inversion point of the system. The wetting index was determined experimentally with the method described above for three different fluid systems. The fluid systems were the Vega condensate without any hydrate inhibitor, Vega with MEG added, and Vega with 1.75 vol % AA, as shown in Table 5. It should be noted that even a positive WI does not guarantee transport without plugging, since other factors also influence, such as AA concentration, slurry viscosity after hydrates are formed, and transport conditions including the degree of turbulence, inclination, low points, and subcooling. These factors must be further evaluated under more realistic flow conditions like flow loops or the wheel flow loop used in the current work.

**Table 5:** Wetting Indices Calculated from Volume Fractions for the Inversion Points with and without Gas Hydrates for the Oil System without and with AA

system	inversion points without gas hydrate particles (water fraction)	inversion points with gas hydrate particles	wetting index
VEGA without AA	0.55	0.35	–0.36
VEGA with 10 wt % MEG on water	0.55	0.45	–0.18
VEGA with 1.75 vol % AA on water	0.85	0.925	+0.5

### 2.5 Wheel Flow Loop Principle and Test Procedure

**Figure 3:**
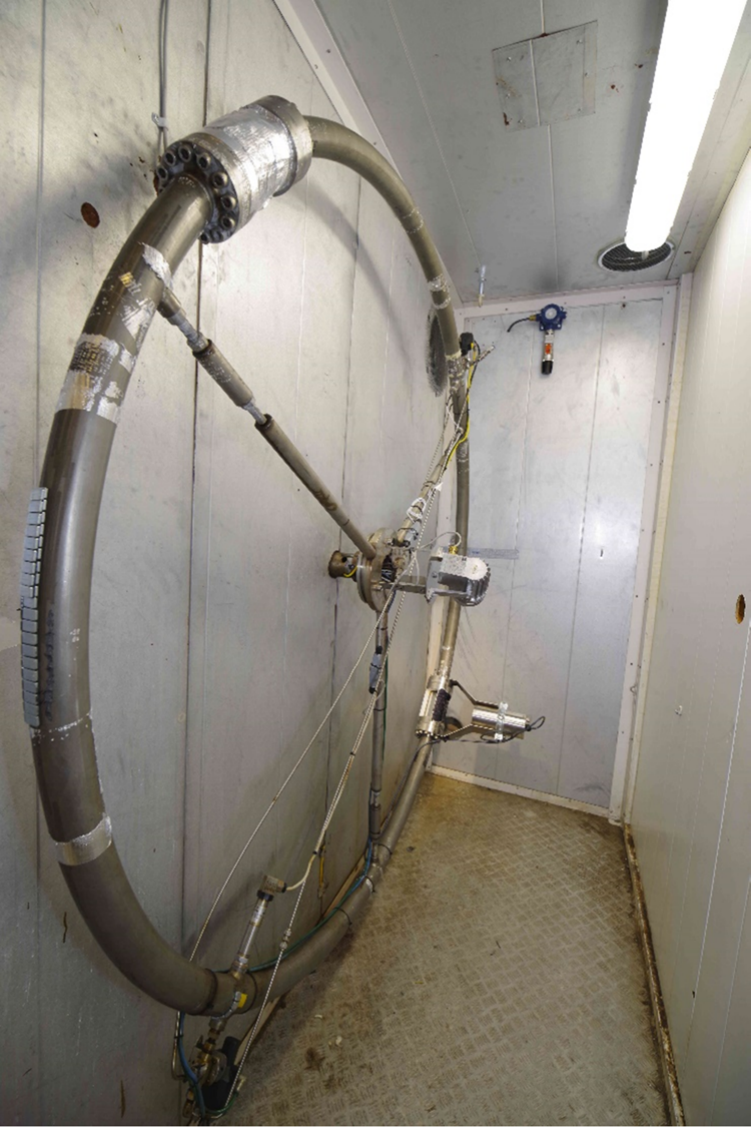
Wheel flow loop. The camera is the white unit sticking out from the wheel farthest away in the picture, close to the floor. It is pointing at the sapphire glass section.

The wheel flow loop, as shown in Figure 3, is a 2″ ID pipe (52.5 mm) shaped into the form of a wheel with an operating pressure of up to 250 bar and located at SINTEF’s Multiphase Flow Laboratory at Tiller in Norway. The wheel was installed in a vertical position so that gravitation keeps the fluids stratified according to their respective densities. Rotation of the wheel creates a relative velocity between the liquids and the pipe wall, leading to friction and a shear force between the pipe wall and the fluid, hence simulating fluid transport through an (infinite) pipeline. Depending on the density difference, viscosities, presence of surface-active components, and rotational velocity, dispersion of the fluids may occur. Furthermore, the wheel is fitted with a sapphire window that enables visual inspection and video recording of the flow behavior throughout the course of the experiments. Hydrate formation can be determined by the pressure and temperature measurements, while the increase in viscosity, deposition, and plugging can be detected by analyzing the data from a torque sensor together with video recordings. The experimental setup is placed inside a temperature-controlled chamber.

The tests in the wheel flow loop were performed to study the effect of the AA concentration on the hydrate slurry transport properties. Furthermore, the water cut was varied to study the effects of the hydrate particle concentration on the slurry transportability. AA concentrations ranged from 0.75 to 3.40 vol % relative to the water, while water cuts ranged between 10 and 59 vol % relative to the liquid content in the wheel flow loop. The values were selected based on suggestions from the AA vendor and field-specific considerations. Further details on the AA concentration and the water cuts are given below.

The wheel experiments consisted of a range of tests at different water cuts, as given in Table 6. The test matrix was chosen to evaluate the effect of AA concentration by testing a range from 0.75 to 3.4 vol % at various water cuts, ranging from 10 to 59 vol %. Furthermore, two dynamic tests at 0.3 and 0.05 m/s velocity were conducted separately with eight shut-in/restart where the wheel was cooled to 4 °C over a period of 12 h, rested for another 12 h, and restarted at 0.3 m/s.

**Table 6:** Overview of the Wheel Tests Performed

test number	water cut	lowest measured temperature	volume % AA relative to water	rotational velocity [m/s]	dynamic or shut-in/restart test	verdict	hydrate fraction in the liquid phase [vol water converted/vol total liquid]	hydrate fraction in the hydrocarbon (HC) phase [vol water converted/vol live HC phase]	max torque before plugging^a^. If no plugging, max torque
#1	63	6.0	0	0.3	dynamic	FAIL/plugging	4.5	10	0
#2	63	6.0	0	0.05	static	FAIL/deposition	19.9	44	0
#3	59	4.0	1.6	0.3	dynamic	FAIL/slurry viscosity	36.5	42.7	25.0
#4	59	4.1	1.6	0.05	dynamic	FAIL/slurry viscosity	0.3^b^	45.3	0.0
#5	59	4.0	1.6	0.3	shut-in/restart	FAIL/slurry viscosity	36.3	36.4	17.6
#6	59	5.1	1.6	0.3	shut-in/restart	FAIL/slurry viscosity	37.9	42.8	25.0
#7	44	5.0	2.2	0.3	shut-in/restart	PASS	33.7	37.7	6.4
#8	29	5.0	3.4	0.3	shut-in/restart	PASS	33.3	21.3	0.2
#9	10	4.0	0.8	0.3	shut-in/restart	FAIL	0.8	5.3	10
#10	10	4.0	1.3	0.3	shut-in/restart	PASS	6.4	6.6	0.2
#11	10	4.0	1.3	0.3	shut-in/restart	PASS	3.8	3.6	0.2
#12	30	4.0	1.5	0.3	shut-in/restart	PASS	19.3	19.4	0.4

a If the verdict was PASS, the value is the maximum torque value measured.

b The wheel did not directly plug at this value, but deposition was observed, which increased over the test period. Deposits at the window indicate the potential for plugging, thus resulting in FAIL.

### 2.6 Calculation of Water Conversion by Hydrate Formation

**Figure 4:**
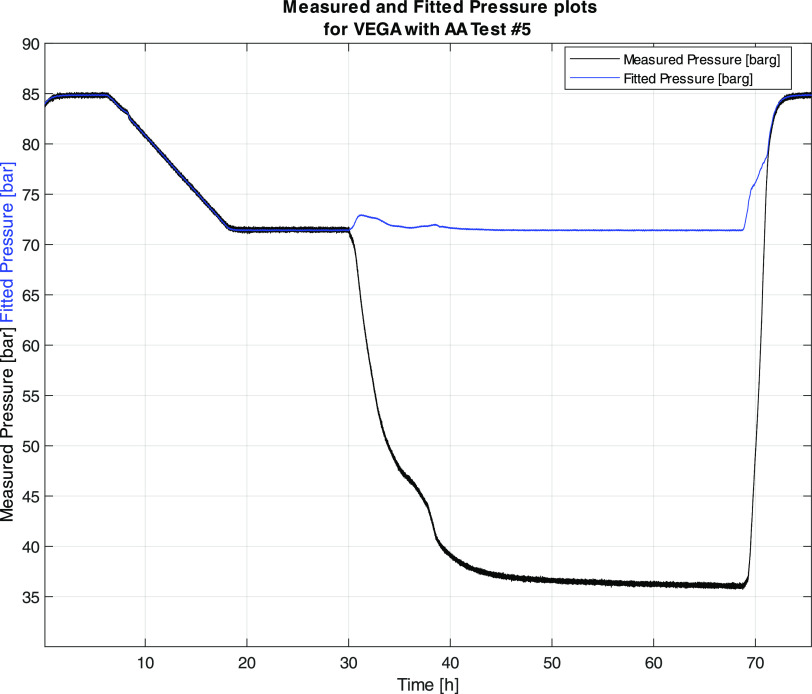
Profiles for the measured and fitted pressures, indicating their deviation when hydrate formation starts at 30 h.

Mole gas in the wheel, *n*, was calculated from the ideal equation of state (EOS), corrected for nonideal behavior using the compressibility factor *z* of the gas phase for the experimental conditions, as shown in eq 3. However, when hydrate formation occurs, the measured pressure is affected by the consumption of gas molecules by the gas hydrate formation process. Thus, a pressure curve, predicted by extrapolation, must be made for the system, representing the pressure relation to the temperature if no gas hydrates were formed. This predicted pressure curve is determined using the relationship between the temperature and pressure prior to hydrate formation and extrapolated for the entire experiment, also where hydrates exist. Any deviation between the experimental pressure and the predicted pressure, Δ*P*, will thus indicate the presence of hydrates and can be used to find the consumption of gas molecules by the hydrate formation. The temperature–pressure relationship is determined by curve fitting in a region just prior to hydrate formation to determine constants *a*, *b*, and *c* in eq 4.
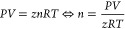
3
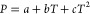
4In eqs 3 and 4, *P* is the pressure, *V* is the volume, *R* is the gas constant, and *T* is the temperature. The result from the extrapolation is illustrated in Figure 4, showing the fitted and measured pressure for Test #5 in Table 6. The difference in actual and predicted pressures is then used for the calculation of Δ*n*. Furthermore, as shown in Figure 4, the predicted pressure fitted well for the system prior to hydrate formation and then started deviating upon hydrate formation, as expected. Then, upon dissociation of the gas hydrates, the measured and fitted pressures reverted back to similar values.

Thus, the amount of consumed gas (Δ*n*) is the difference between the expected amount of gas in the gas phase (*n*_expected_), calculated from extrapolating the EOS, and the measured amount of gas (*n*_measured_), calculated from the experimental data, as given in eq 5
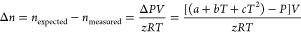
5Moreover, from Δ*n*, the amount of water converted to gas hydrates and the volume % of hydrates in the liquid phase can be calculated. The conversion from mole gas consumed to weight water consumed by hydrate formation is based on assumptions of the percentage of cavities in the hydrate structure that are actually occupied by gas molecules. These numbers have been reported to be higher than 95% for the large cavities and approximately 50% for the small cavities (eq 6).^1^ For structure II hydrates, as will be formed by the gas composition used, there will be 8 large cavities, 16 small cavities, and 136 molecules in a unit cell, resulting in the number of moles of gas per unit cell.
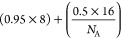
6giving eq 7
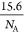
7where *N*_A_ is Avogadro’s number. Furthermore, the relative gas to water in the hydrates is given by eq 8.
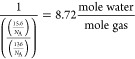
8

## 3 Results and Discussion

### 3.1 Wettability Modification of Gas Hydrates by Addition of AA

The WI indices determined for systems without AA, with 10 wt % MEG, and with 1.75 vol % AA are given in Table 5. The addition of AA improved the wetting index from a negative value (−0.36) for the uninhibited Vega condensate to a positive value (+0.5) for the AA-inhibited system. By this improvement, it is indicated that a positive WI number indicates a higher probability for a transportable hydrate slurry system. Thus, the selected AA, and AAs with equivalent properties, should be able to produce a dispersed and transportable slurry phase as long as the slurry viscosity is within the limits for flowability, which is dependent on the driving forces and geometries of the production facilities. Addition of 10 wt % MEG increased the WI index to −0.18, which still indicates water-wet hydrate particles and potential for hydrate plugging. The addition of MEG was, however, not expected to turn the system into a nonplugging one with the presence of hydrates, as MEGs’ main effect is to reduce the equilibrium temperature for the hydrate curve. Despite the positive WI for the AA system, it does not indicate the concentration of the inhibitor needed to assure pipeline transport without plugging. Therefore, the wheel flow loop experiments were performed at a range of AA concentrations for several relevant water cuts. The combined results may help indicate the applicability and efficiency of the AA used or the comparison of several LDHIs.

**Figure 5:**
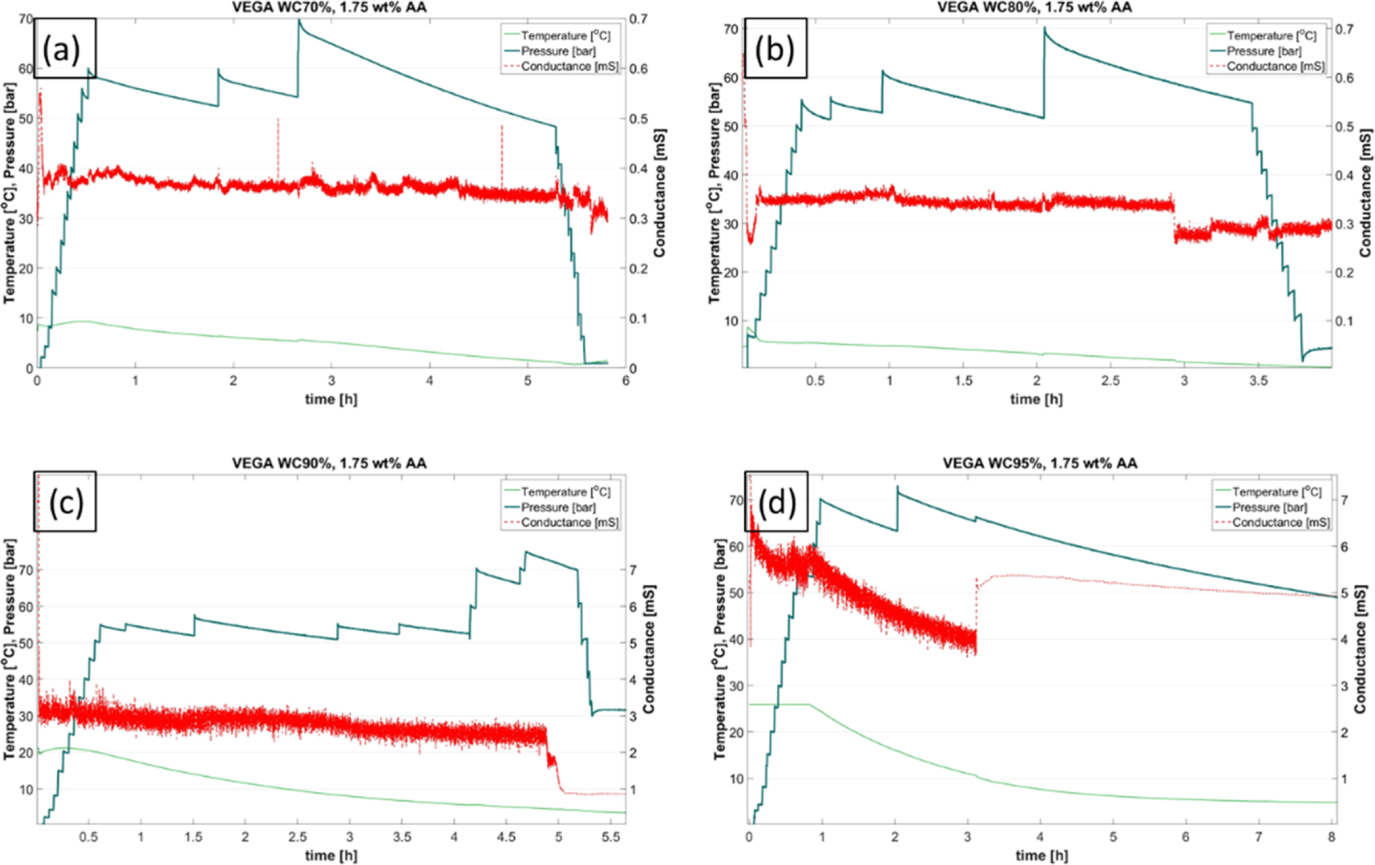
Plots from the individual tests for four water cuts (a)–(d) with water cuts of 70, 80, 90, and 95%, respectively, for Vega with 1.75 vol % AA. The red plot is the conductance and indicates low conductivity before and after hydrate formation (∼0.3 mS) up to WC80 (a and b). At WC90 (c), the conductance was high before hydrate formation and drops upon hydrate formation, and for WC95 (d), the conductance is high both before and after hydrate formation.

**Figure 6:**
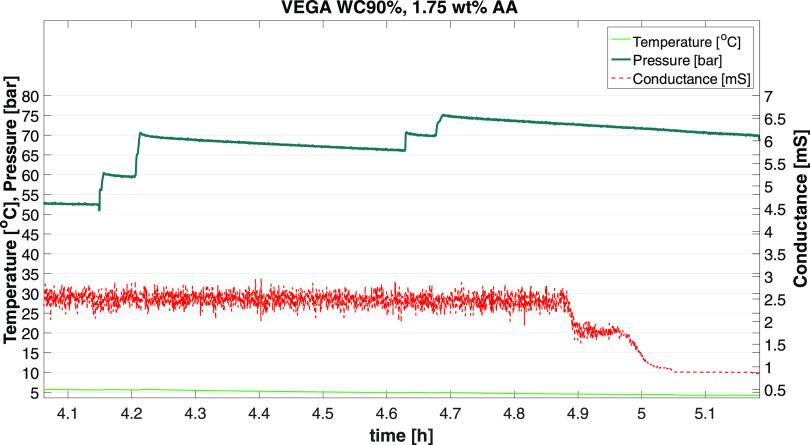
Temperature, pressure, and conductance for the WI test performed at WC90 with 1.75 vol % AA. This plot shows the conductance dropping at a point where there were no changes in the pressure or temperature (at time 4.8–5.0 h), indicating the occurrence of hydrate formation.

**Figure 7:**
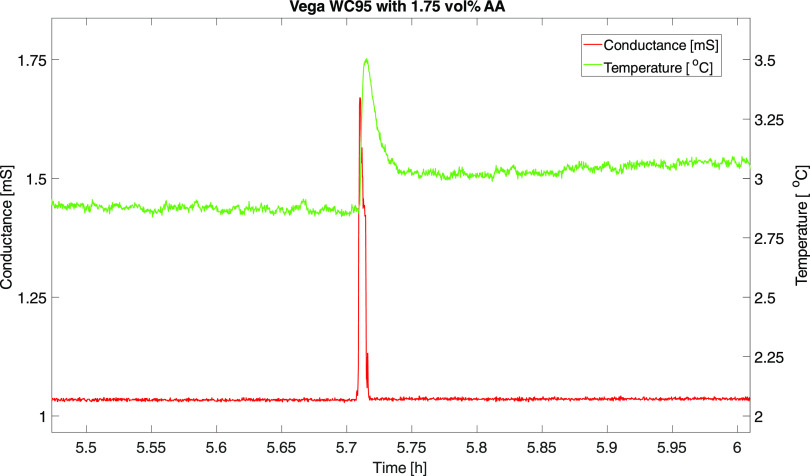
Zoom in on the time of the hydrate formation, indicating the temperature increase upon hydrate formation. Also, the conductance spiked slightly, indicating a temporary change in the conductance during the initial phase of the hydrate formation.

Interpretation of the WI indices was based on the measured conductance before and after hydrate formation following the procedure described above, as exemplified by plots in Figure 5, showing the measured temperature, pressure, and conductance when cooled into the hydrate region. The conductance for the water cuts up to 80 vol % (plots a and b in Figure 5) was low, with a value fluctuating between 0.3 and 0.4 mS indicating oil continuous systems. When the WC was increased to 90 vol % (plot c in Figure 5), the measured conductance was much higher (between 2 and 3 mS) before the hydrates were formed and dropped to 0.85 mS, indicating a transition from a water continuous system to an oil continuous system upon hydrate formation. To achieve a water continuous system both before and after hydrate formation, a WC of 95 vol % was necessary, as indicated by plot d in Figure 5. Figure 6 shows a zoom-in view of the hydrate formation region for WC90 to show that the hydrate formation (determined by the drop in conductance) occurred before the pressure reduction occurred. Furthermore, the exothermic reaction of hydrate formation can often be detected from the temperature profile from the WC95 vol % water cut, as shown in Figure 7. However, for the water cuts below WC90, such temperature increases were not detected from the data, which is not uncommon for oil continuous systems or when inversion to oil continuous systems occurs. This is due to the lower heat conductivity of the oil phase. Therefore, visual inspection was required to verify hydrate formation.

### 3.2 Experimental Quantification of AA Dosage Using Flow Loop Experiments

**Figure 8:**
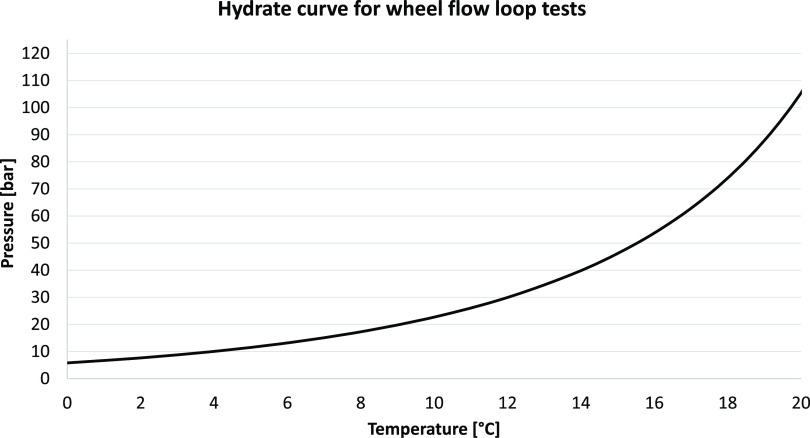
Hydrate curve for the filling composition to the wheel flow loop.

The “minimum effective dosage” (MED) represents the minimum necessary amount of AA to protect a given fluid system. MED can be determined by testing various AA concentrations for the same fluid system, including water cut. Table 6 shows the results from the flow loop tests in terms of information on water cut, AA concentration, the verdict of each test, the amount of water converted by hydrate formation, hydrate fraction relative to the hydrocarbon phase, and the maximum torque. For the torque value, if the verdict was “FAIL,” the reported value would be the maximum torque value observed before plugging occurred. Baseline tests at 63 vol % water cut without any AA were conducted and resulted in plugging. Then, the highest water cuts run with AA were 59 vol % with an AA concentration of 1.6 vol %. The general observation was that the system formed gas hydrates that initially were transportable, but eventually plugged. The reason for plugging could be the increased viscosity of the hydrate slurry making transportability inside the 2″ pipe of the wheel not possible. However, it may also be due to the underdosing of AA making the system protected only up to a given amount of hydrates formed. The type of data and results obtained by the wheel are not achievable by stirring cell, indicating the relevance of the combined use of WI and flow loop studies. As indicated in Table 6, tests based on relevant field-specific cases with WC“*x*” and AA“*y*” concentrations of WC44/AA2.2, WC29/AA3.4, WC10/AA0.75, and WC10/AA1.25 were conducted. Of these, only the lowest AA concentration of 0.75 vol % resulted in a failure to disperse the hydrates and transport the gas hydrates within the liquid phase without agglomeration. Below, selected results from the baseline tests and the tests with AA are presented to show the interpretation of the flow loop tests and the evaluation of the transportability of the hydrate slurries formed. The gas hydrate curve for the filling composition to the wheel flow loop is given in Figure 8 and mimics the hydrate curve for the reservoir fluid compositions available. The figure indicates the subcooling upon hydrate formation for the various tests ranging from around 11 to 13 °C at a pressure of 70 bar, which was the pressure upon hydrate formation. Nevertheless, for a system well inside the hydrate region, smaller variations in the subcooling, a few °C, will not have a very large effect on the behavior of the AA as long as the availability of gas for hydrate formation is sufficient. This may of course also depend on the AA’s overall solubility and chemistry, which may be affected by temperature.

#### 3.2.1 Baseline Tests with an Unprotected System (Tests #1 and #2)

Two baseline tests were conducted to establish the hydrate transport properties for an unprotected fluid system. They consisted of a dynamic cooling test, where the system was cooled into the hydrate region while transporting at a velocity of 0.3 m/s. The second test consisted of shut-in/restart, where the rotation was stopped at an elevated temperature and then cooled into the hydrate region of the system before restarting at 0.05 m/s. The reason for the lower velocity for the second baseline test was to reduce the rate of hydrate formation and possibly avoid plugging.

**Figure 9:**
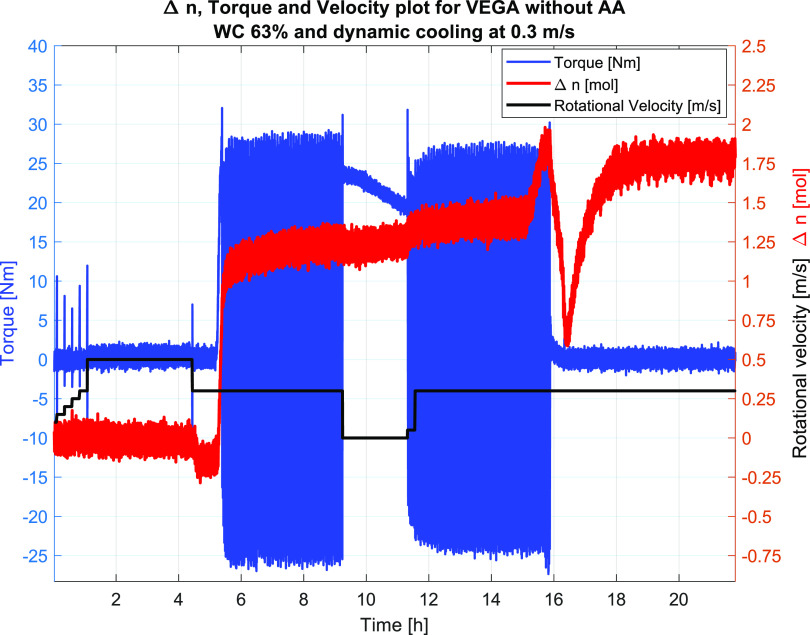
Test #1, the plot shows the rotational velocity, torque values, and Δ*n*. The torque values indicate the plugging of the wheel flow loop upon hydrate formation when no AA is present.

The results in terms of mole gas consumed by hydrate formation, the torque values, and the velocity profiles are shown in Figure 9 for Test #1 and in Figure 12 for Test #2. Plugging of the flow loop occurred almost immediately for both systems (around 9 and 8 min) upon hydrate formation. Δ*n* due to hydrate formation was 1.8 mol for Test #1 or 269 g of water when converted using eq 8, representing an approximate volume fraction of 8.5 vol % gas hydrates relative to the water volume and 5% relative to the liquid volume. For Test #2, the mole gas converted by hydrate formation was more than double (4 mol) that for Test #1, giving a hydrate fraction of close to 20 vol %. The reason for the difference in Δ*n* could be the different velocities, where the higher velocity would give faster initial hydrate formation but would stop due to major plugs, restricting the access of free gas to the water phase in the sections of the wheel and thus stopping the hydrate formation. For the lower velocity (0.05 m/s), the mixing would be less and the hydrates, although stuck to the wall, would take a longer time to plug the entire wheel, thus giving a longer time for hydrate formation and more hydrates to be formed, as indicated by the Δ*n* plot in Figure 12, showing a less steep slope than that for Test #1. An intermediate shut-in period (from 10 to 12.2 h in the plot in Figure 9) was tested for Test #1 and showed a gradual reduction in the torque values from 23 to 19 Nm, indicating some movement of the hydrate phase inside the wheel or gradually draining of the liquid trapped above a semipermeable plug, thus shifting the balance of the wheel. Upon restarting at 0.3 m/s, the wheel was still plugged. Furthermore, for Test #1, the hydrate plug did not let go (loosen from the walls inside the wheel) until it was melted at 17.2 h experiment time.

**Figure 10:**
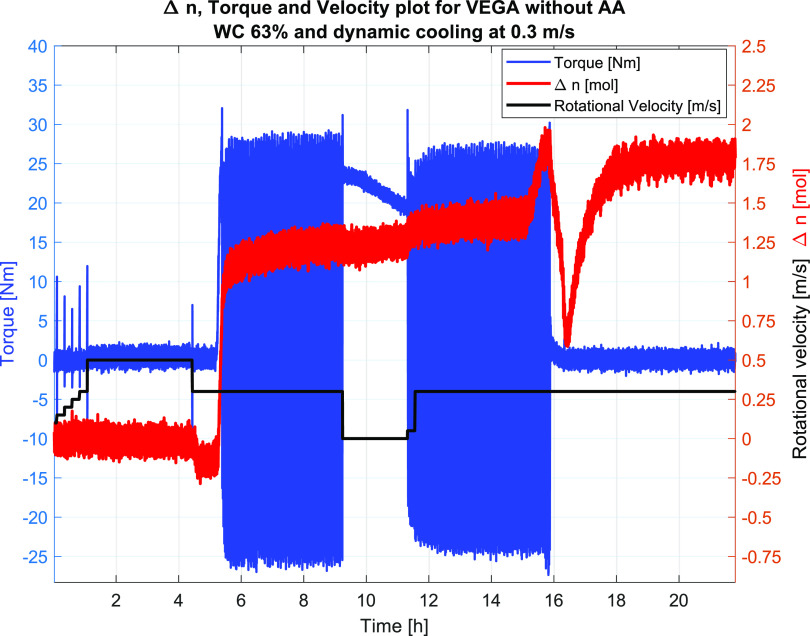
Plot of pressure and temperature versus time for Test #1.

Figure 10 shows the pressure and temperature profiles from Test #1, indicating the pressure correlation with temperature until hydrate formation occurred at 5.7 h after which the pressure profile reduction became steeper and the temperature increased due to the exothermic reaction of hydrate formation. Furthermore, one can also observe a small reduction in the pressure, while the temperature stayed constant, indicating hydrate formation throughout the test duration.

**Figure 11:**
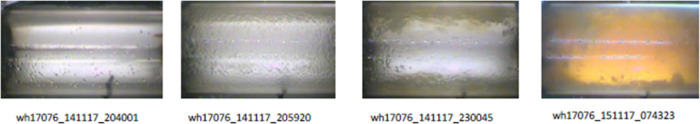
Test #1, pictures of the sapphire window at various times. The text below the pictures indicates the instrument test ID and the date and time when the picture was taken, giving a general time stamp of “*whxxxx ddmmyy_hhmmss*”.

**Figure 12:**
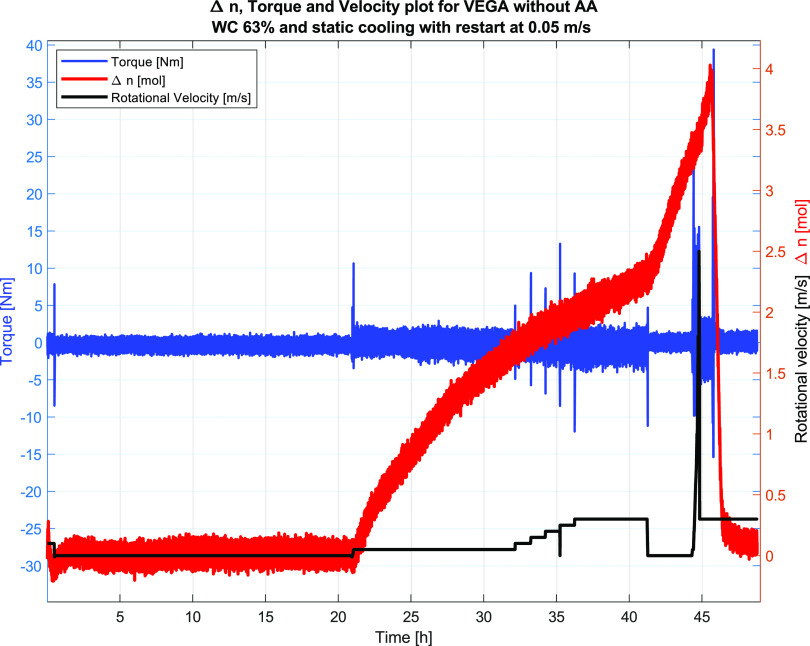
Test #2 at 63% WC showing the torque values, velocity, and Δ*n*. Also here, the torque data indicated plugging upon hydrate formation. Restart is observed at ∼21 h after which the hydrate formation starts and continues throughout the test period. The torque data indicate plugging, since they fluctuate around zero.

The picture series given in Figure 11 shows the fluids through the sapphire window of the wheel flow loop for Test #1. The first picture from the left is of the window in the gas phase before hydrate formation, the second and third pictures show hydrates deposited at the window, and the rightmost picture shows hydrates in the liquid phase under plugging conditions. Furthermore, as indicated above, Figure 12 below shows the Torque, Δ*n* and velocity profile for Test #2.

**Figure 13:**
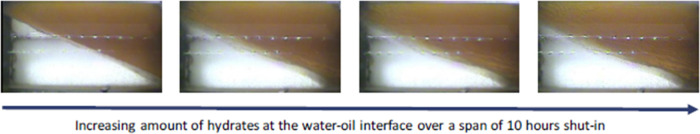
Test #2, from left to right, this picture series shows hydrate growth at the water–oil interface.

The picture series given in Figure 13, from Test #2, shows the water–oil interface over a span of 10 h under stagnant conditions. From left to right, the picture series shows hydrate growth at the interface.

**Figure 14:**
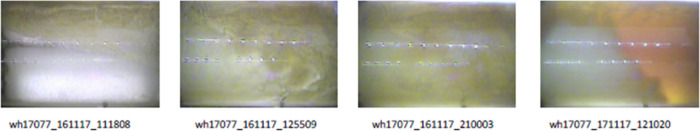
Test #2, this picture series shows, for the first three from the left, increasing amounts of hydrates deposited at the wall. The rightmost picture shows a lump of hydrate (white mass to the left) in the oil phase during melting.

Figure 14 shows, for the first three pictures from the left, increasing amounts of hydrates deposited at the wall for Test #2 during hydrate formation. The rightmost picture shows a lump of hydrate (white mass to the left) in the oil phase during melting.

#### 3.2.2 Flow Loop Tests with Underinhibited Systems Exemplified with Test #4

**Figure 15:**
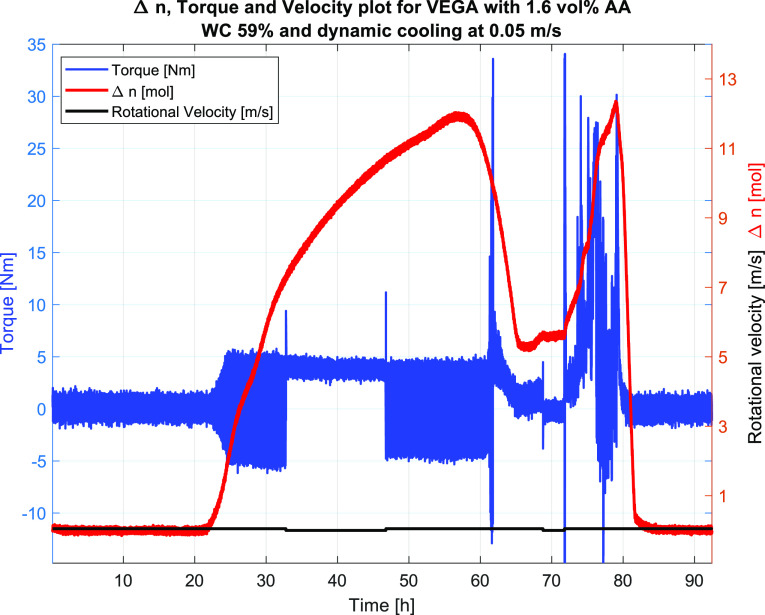
Test #4, the plot shows torque, velocity, and Δ*n*. The torque data indicate plugging upon hydrate formation.

**Figure 16:**
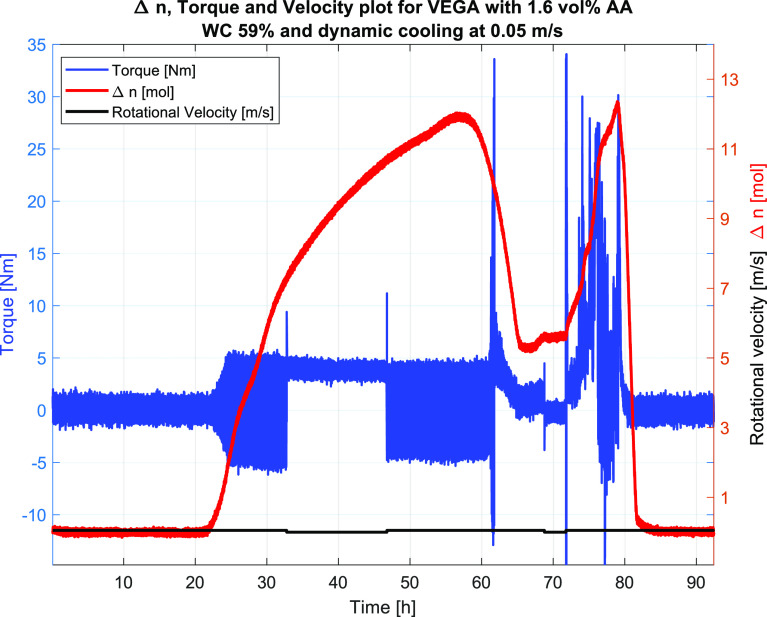
Test #4, the plot shows temperature and pressure profiles. Hydrate formation is indicated by the increase in temperature at around 21 h, correlating with a change in the slope of the pressure profile.

Fluid systems containing a relatively low concentration of AA of 1.6 vol % relative to water with a water cut of 59 vol % were exposed to various test procedures, as indicated in Table 6 (Tests #3–#6). The AA concentration could, based on information from the vendor, have the potential to protect the current fluid system. However, for all of the tests, plugging after hydrate formation occurred. Below are the results from two of the tests (Tests #4 and #5) discussed. For Test #4, the system cooled into the hydrate region while rotating at 0.05 m/s. Furthermore, a 12 h shut-in was conducted after hydrate formation and plugging had occurred. The following restart at 0.05 m/s showed that the wheel was still plugged. Then, a partial melting procedure increased the temperature gradually to 16 °C over a 12 h period while rotating at 0.05 m/s, thus partly melting the hydrates, as indicated by the Δ*n* values not going to zero (Figure 15). The temperature and pressure profiles for this test are given in Figure 16. The result of this reheating was that the hydrates became transportable at around 62 h when the temperature was 12 °C but plugged again after cooling to 4 °C; the system re-forming hydrates indicated that the plugging was due to either increasing slurry viscosity as the amount of hydrate particles grew or underdosing so that the AA protection was only sufficient for a given water conversion, as indicated by the mole gas conversion (Δ*n*). Although this shut-in procedure was conducted for Test #4 but not for Test #5, it still indicated the effect of AA to make the hydrates more transportable for the Vega fluid system and may indicate that a concentration of 1.6 vol % was at the borderline of MED.

**Figure 17:**
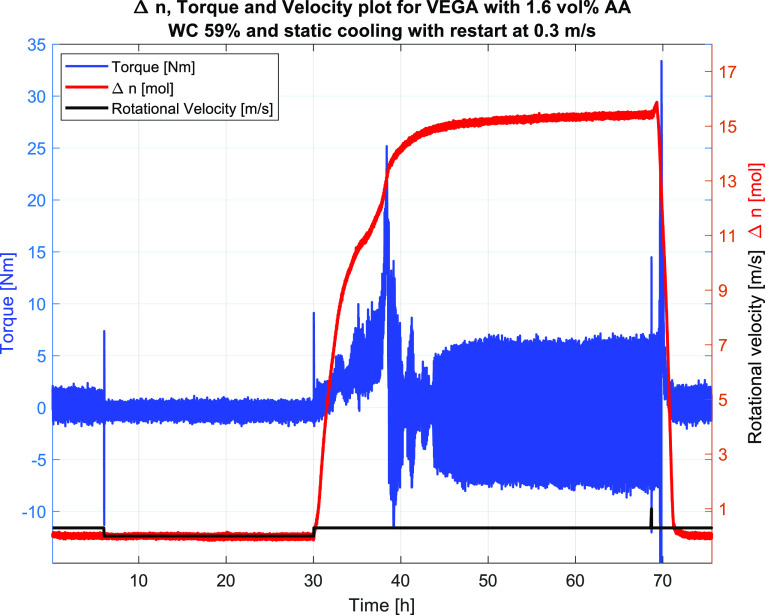
Test #5, the plot of raw torque, velocity, and Δ*n*, indicating hydrate formation occurring at 30 h into the experiment and plugging occurring at around 38 h into the experiment.

**Figure 18:**
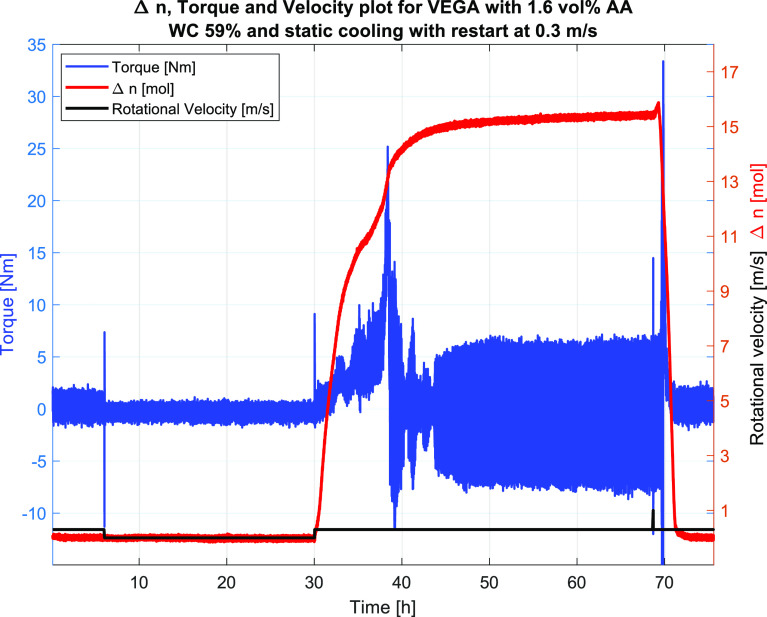
Test #5, the plot shows the temperature and pressure profiles. Hydrate formation is indicated by the increase in temperature at around 21 h, correlating with a change in the slope of the pressure profile.

As Test #4 was run at 0.05 m/s dynamic cooling into the hydrate region and plugged upon hydrate formation, Test #5 was shut-in at 35 °C and cooled into the hydrate region before restarting at 0.3 m/s. This test did not plug until 8 h after hydrate formation at 38 h (Figure 17), indicating the effect of the AA up to a certain point, after which the AA may have been consumed leaving the remaining hydrates unprotected and thus susceptible to plugging, as discussed for Test #4 above. This shows the need for testing inhibitors at relevant concentrations under more realistic conditions than those available in stirring cells and similar small volumes without pipe flow conditions. The temperature and pressure profiles for Test #5 are given in Figure 18, indicating a significant increase in temperature upon hydrate formation at 30 h.

It was observed that the systems containing AA allowed much more hydrate to be formed, even when plugged, compared to the nonprotected system, indicating that the plugging may restrict the overall hydrate formation.

#### 3.2.3 Fully Inhibited System Exemplified with Test #7

**Figure 19:**
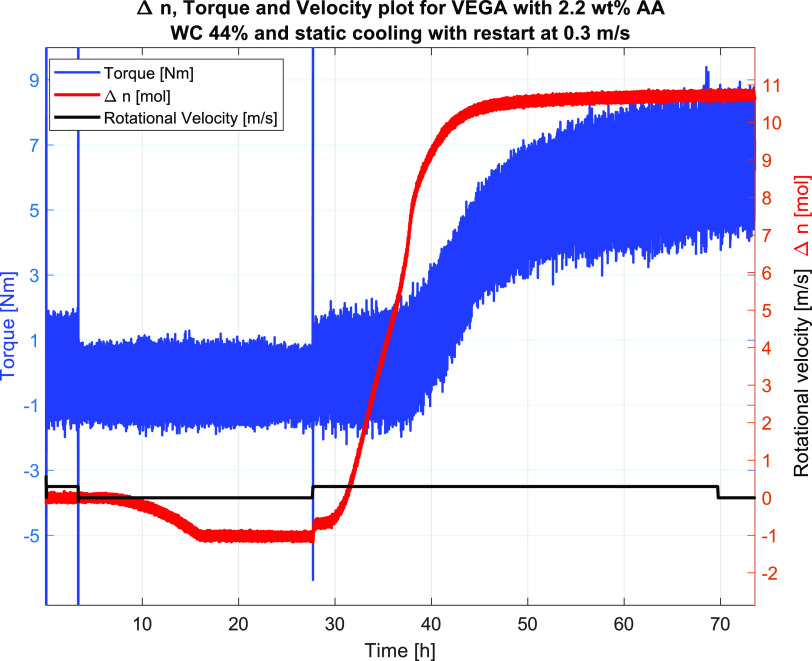
Test #7, the plot shows Δ*n*, raw torque data, and velocity.

**Figure 20:**
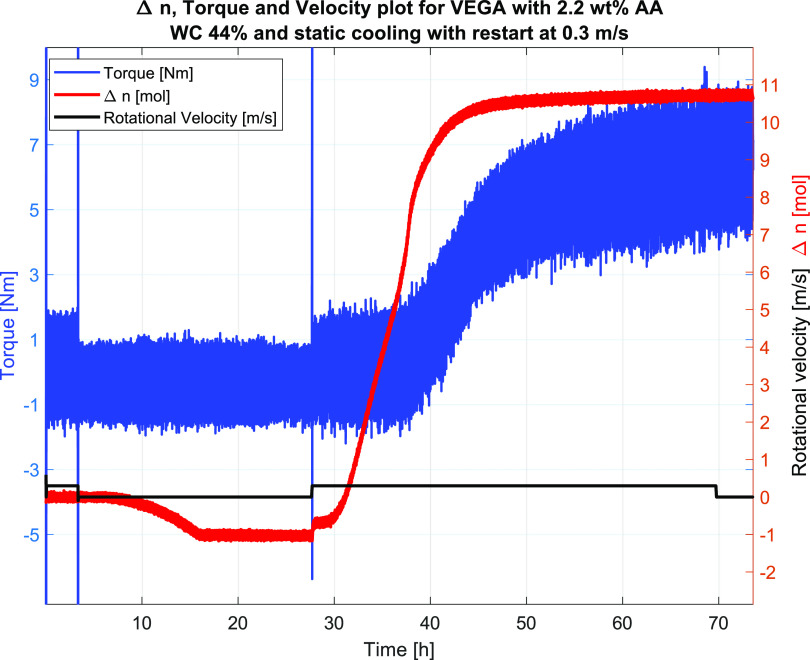
Test #7, the plot shows the temperature and pressure profiles. Hydrate formation was detected by the temperature increase correlating with the pressure drop upon restart of rotation.

Tests that indicated effective AA concentrations, indicated by the results from the flow loop tests, were Tests #7–#12. Their water cuts and AA concentrations as well as other test information and results are given in Table 6. Selected for a more detailed presentation was Test #7 with a WC of 44 vol % and an AA concentration of 2.2 vol %. The results indicated that the system now had become fully protected against plugging at the experimental conditions with the test facilities used. Results in terms of Δ*n*, torque, and velocity profiles for Test #7 are given in Figure 19, while the pressure and temperature profiles are given in Figure 20. The data clearly showed that the temperature increase correlated with the pressure drop upon hydrate formation at 30 h. Moreover, the torque values increased gradually and smoothly upon increasing hydrate formation. Thus, the increased AA concentration managed to protect the system at the given water cut. The Δ*n* profile dropped below zero during the cooling period, as shown in Figure 19. This is an artifact that sometimes occurs during the calculation of shut-in systems due to the nonequilibrium of the gas–oil equilibrium and does not affect the overall results or interpretations. Furthermore, the max Δ*n* values are comparable to the ones at lower AA concentrations (Tests #4 and #5), indicating that the same amounts of hydrates can lead to both plugging and a transportable system depending on how well it is protected by the AA. From the current results, one can say that the wheel flow loop is a very convenient tool for evaluating the degree of protection for a given fluid system. However, one needs to evaluate also other relevant field conditions based on the lab results.

To summarize the results from the flow loop tests, the relation between the AA concentration and the water cut was a factor in terms of protecting the system against plugging. At 10% WC, an AA concentration of 1.25 vol % was sufficient to protect against hydrate formation, while for higher (∼60 vol %) water cuts, AA concentrations between 1.6 and 2.2 vol % were necessary to protect against hydrate plugging. This shows that studies to determine the appropriate dosage of AA should be performed under conditions as close as possible to the given field, including oil and gas fractions, water chemistry, and the water cut. Nevertheless, laboratory-obtained results should also be treated with care when used as input to decisions on the operation of oil and gas fields.

## 6 Conclusions

The experiments performed were conducted to show the complementary gain when determining both the wetting and flow properties of hydrate systems at different degrees of protection. The system selected to show this was a specific, field-relevant, condensate system with known plugging issues when not protected. However, irrespective of the type of antiagglomerant (AA) if it is working as intended, the overall results should be comparable to the ones reported here. Moreover, three different production scenarios were discussed with regard to the risk for hydrate plugging of the flowline, consisting of (1) no active inhibition, (2) 10% MEG added, and (3) the use of AA. The three scenarios were experimentally evaluated using SINTEF’s wetting index cell, while only unprotected and AA-protected fluid systems were further studied with the wheel flow loop. The wetting index cell was utilized to determine the wettability and thus the potential for hydrate plugging by interpreting the difference in the emulsion inversion point with and without gas hydrates for the three scenarios. At an AA concentration of 1.75 vol %, WI for the fluid system was measured to be +0.5. In comparison, the uninhibited system had a WI of −0.36, and with MEG, the WI was −0.18. The results thus indicated that the addition of the AA should result in a dispersed hydrate phase with a lower plugging risk than the unprotected system.

The conclusion of the wheel flow loop test was that the addition of 1.6 vol % AA was insufficient to fully protect against plugging at 59 vol % WC, while 2.2 vol % did protect against plugging for 44 vol % WC. At a WC of 10 vol %, an AA concentration of 0.8 vol % was not enough to protect against plugging, while 1.3 vol % AA was. This indicates the need for experimental data on inhibitor dosage for a given fluid system. Moreover, the results show that AA addition must be evaluated at the relevant water cuts, since it seemed like higher water cuts would require more AA relative to water. This further indicates the need for improved methods for predicting AA addition considering the water cut.

For a condensate system, like the Vega asset, the current results and evaluations emphasize the importance of an active hydrate mitigation approach, either in moving out of the hydrate window by depressurization or by addition of a suitable AA. The risk of creating hydrate plugs is just too high in case such a system is left deep within the hydrate region. The change in the wetting index by the addition of 10% MEG did not result in a significantly larger safe operational envelope. For the tested conditions, an AA appears to be a feasible technical solution to the challenge, but requirements on both the environmental and economic sides would need further consideration before moving into regular field applications.

## Abbreviations

AA: antiagglomerant
WC: water cut
WI: wetting index
